# A Serpin With a Finger in Many PAIs: PAI-1's Central Function in Thromboinflammation and Cardiovascular Disease

**DOI:** 10.3389/fcvm.2021.653655

**Published:** 2021-04-16

**Authors:** Gael B. Morrow, Claire S. Whyte, Nicola J. Mutch

**Affiliations:** ^1^Aberdeen Cardiovascular and Diabetes Centre, Institute of Medical Sciences, School of Medicine, Medical Sciences and Nutrition, University of Aberdeen, Aberdeen, United Kingdom; ^2^Radcliffe Department of Medicine, University of Oxford, Oxford, United Kingdom

**Keywords:** PAI-1, fibrinolysis, metabolic syndrome, obese, diabetes, thrombosis, inflammation

## Abstract

Plasminogen activator inhibitor 1 (PAI-1) is a member of the serine protease inhibitor (serpin) superfamily. PAI-1 is the principal inhibitor of the plasminogen activators, tissue plasminogen activator (tPA), and urokinase-type plasminogen activator (uPA). Turbulence in the levels of PAI-1 tilts the balance of the hemostatic system resulting in bleeding or thrombotic complications. Not surprisingly, there is strong evidence that documents the role of PAI-1 in cardiovascular disease. The more recent uncovering of the coalition between the hemostatic and inflammatory pathways has exposed a distinct role for PAI-1. The storm of proinflammatory cytokines liberated during inflammation, including IL-6 and TNF-α, directly influence PAI-1 synthesis and increase circulating levels of this serpin. Consequently, elevated levels of PAI-1 are commonplace during infection and are frequently associated with a hypofibrinolytic state and thrombotic complications. Elevated PAI-1 levels are also a feature of metabolic syndrome, which is defined by a cluster of abnormalities including obesity, type 2 diabetes, hypertension, and elevated triglyceride. Metabolic syndrome is in itself defined as a proinflammatory state associated with elevated levels of cytokines. In addition, insulin has a direct impact on PAI-1 synthesis bridging these pathways. This review describes the key physiological functions of PAI-1 and how these become perturbed during disease processes. We focus on the direct relationship between PAI-1 and inflammation and the repercussion in terms of an ensuing hypofibrinolytic state and thromboembolic complications. Collectively, these observations strengthen the utility of PAI-1 as a viable drug target for the treatment of various diseases.

## Introduction

Plasminogen activator inhibitor-1 (PAI-1) is a fast-acting serpin that regulates the fibrinolytic system through inhibition of tissue plasminogen activator (tPA) and urokinase-type plasminogen activator (uPA). PAI-1 quenches the enzymatic activity of these proteases to constrain fibrin degradation and stabilize the hemostatic plug. Like other serpins, PAI-1 forms a 1:1 enzyme-inhibitor complex with its target proteases, rendering them enzymatically inactive and resulting in rapid clearance from the circulation *via* the hepatic system. However, PAI-1 is an unusual serpin in that it can lose activity by spontaneous insertion of the reactive center loop into the body of the molecule, forming “latent” PAI-1 (1). The active form of PAI-1 is very unstable and has a short half-life of 1 h (2), whereas conversion to its thermodynamically stable latent form allows a prolonged half-life of 2–4 h (3). In healthy individuals' plasma, PAI-1 circulates in excess over its target protease tPA but at relatively low concentrations compared with other serpins, and is highly variable in normal individuals (1–40 ng/ml). PAI-1 is relatively unstable with a half-life of around 1–2 h in the circulation (4), however, binding to the extracellular matrix protein, vitronectin, stabilizes the active form of PAI-1 (5), and augments its half-life (6).

Circulating PAI-1 levels are under genetic control that is directly related to an insertion/deletion (5G/4G) polymorphism at position −675 of the promoter (7). The 4G allele gives rise to elevated plasma PAI-1 levels (8–11). PAI-1 displays circadian rhythm with a peak in early morning that coincides with the time of onset of myocardial infarction (MI) (12). The 4G/5G polymorphism differs according to ethnic group which has a direct impact on PAI-1 circulating levels (13). Interestingly, the levels of PAI-1 vary according to gender and show a positive correlation with increasing age (14).

**Figure 1:**
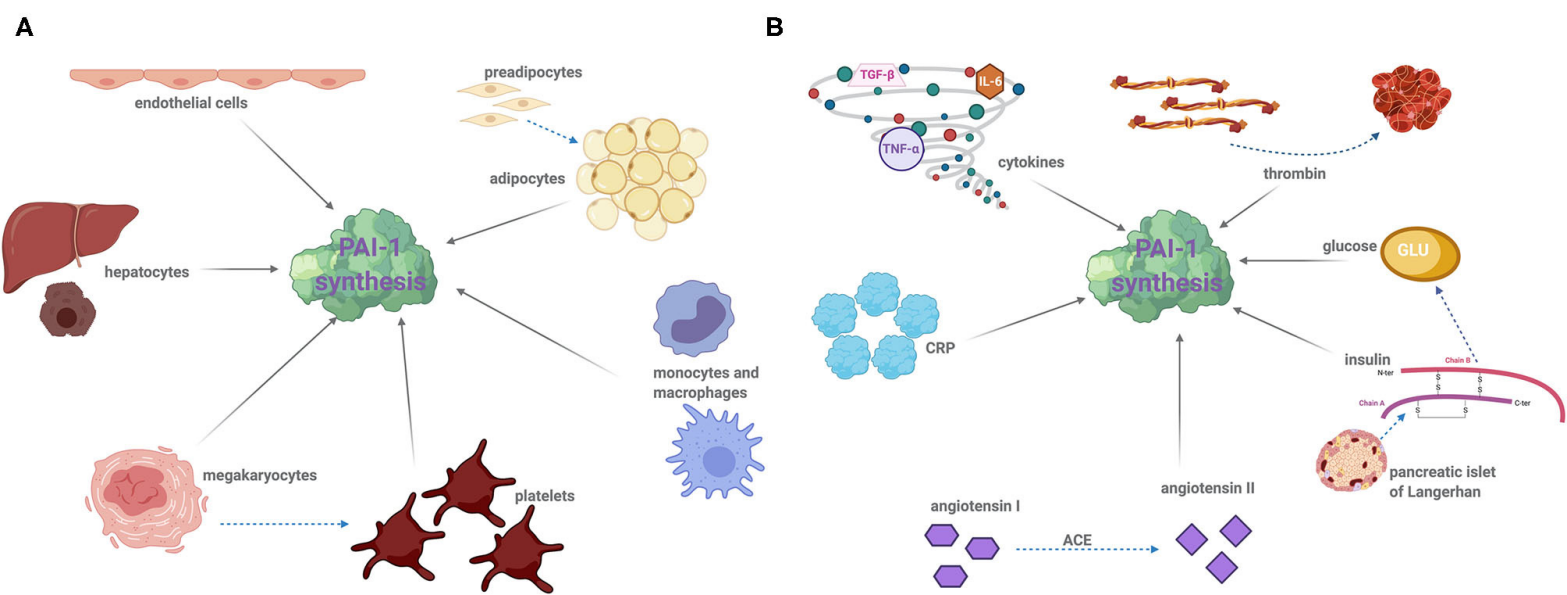
Cellular sources of PAI-1 of modulators of synthesis: **(A)** PAI-1 is produced by a variety of cell types including the following: endothelial cells, adipocytes, hepatocytes, leukocytes (monocytes and macrophages), megakaryocytes, and platelets. Dotted arrows demonstrated cell differentiation and solid arrows indicate PAI-1 synthesis. **(B)** A number of factors induce PAI-1 synthesis and secretion. Proinflammatory cytokines, namely interleukin-6 (IL-6), tissue necrosis factor alpha (TNF-α), and transforming growth factor beta (TGF-β) significantly augment PAI-1 synthesis. Thrombin, insulin, glucose, angiotensin II, and C-reactive protein (CRP) can also stimulate PAI-1 expression. Drugs that target insulin and the angiotensin converting enzyme (ACE), such as metformin and captopril, are known to decrease plasma PAI-1 concentration. Dotted arrows represent the reaction of an enzyme or protein synthesis, and solid arrows indicate PAI-1 synthesis.

Platelets contain the major pool of circulating PAI-1, which when activated following vessel injury, release this cargo thereby protecting the developing thrombus from premature fibrinolysis. Not surprisingly, the platelet precursor cell, megakaryocytes, are a major site of PAI-1 synthesis (15) and platelets themselves are now known to retain some PAI-1 mRNA which can produce functional protein (16). However, PAI-1 is also synthesized by other cells including endothelial (17), adipocytes (18–20), hepatocytes (21), and cardiomyocytes (22) (Figure 1A). Given the crucial role of PAI-1 in hemostasis, a deficiency in this serpin gives rise to a moderate bleeding diathesis (23). Conversely, increased levels of PAI-1 are associated with thrombotic complications. In addition to its hemostatic role, PAI-1 functions in several physiological processes such as inflammation, wound healing, and tumor progression. A strong relationship between PAI-1 and obesity, diabetes, and metabolic syndrome (MetS) was recognized many years ago with this serpin now being considered central to these pathophysiological processes (24). This review will focus on the impact and relationship of this unusual serpin in dictating and orchestrating the development of thromboinflammation and cardiovascular complications as a result of its participation in the pathogenesis of associated diseases.

## PAI-1 and Thrombosis

The fate of a forming thrombus is determined by platelet deposition and the balance of coagulation and fibrinolytic factors. An increase in circulating levels of PAI-1 or augmented local release of this inhibitor due to platelet activation shifts the balance to a hypofibrinolytic state. PAI-1 has been recognized as a pivotal protein in the progression of vascular events and is linked to MI (25–27), stroke (28), deep vein thrombosis (DVT) (29), and microvascular thrombosis (30). Elevated levels of plasma PAI-1 precede the occurrence of MI (26), and survivors exhibit consistently high levels (25). Acute increases in plasma PAI-1 levels within 24 h in patients with acute ST-elevated myocardial infarction are associated with heart failure and death and are a strong independent predictor of mortality at 30 days (31). The renin-angiotensin II system (RAS) is strongly activated following acute MI, and angiotensin II triggers PAI-1 synthesis (32) (Figure 1B). The RAS has been linked to the circadian variation in PAI-1, suggesting that the use of angiotensin-converting enzyme (ACE) inhibitors to inhibit the RAS system may blunt PAI-1 levels thereby reducing the risk of “early morning” MI (33).

Elevated levels of PAI-1 have been detected in atherosclerotic plaques in humans (34–36), which are significantly inflated in type 2 diabetes mellitus (T2DM) subjects (37). Dysregulation of the fibrinolytic system appears to play a significant role in atherosclerotic plaque development by perturbing the wound-healing response and neointimal formation (38). This can in part be attributed to reduced vascular smooth muscle cell migration *via* inhibition of binding of vitronectin to integrin α_*v*_β_3_ (39). Moreover, PAI-1 stabilizes the fibrin matrix within the developing plaque by attenuating plasmin formation. Increased serum levels of PAI-1 have been noted in patients with atherosclerotic disease, including coronary artery disease (40) and stroke (41). A recent large meta-analysis has indicated that PAI-1 is implicated in the pathogenesis of atherosclerotic disease (42). The two-pronged approach of PAI-1 inducing a hypofibrinolytic state in atherosclerotic diseases and impacting on lesion formation and progression indicates the perilous complications of this serpin in the pathogenesis of these diseases. Animal models of ablation (43, 44) of the PAI-1 gene and pharmacological inhibition of PAI-1 (45) in murine models have proved insightful in our understanding of these processes. Nevertheless, it is challenging to extrapolate all of these findings to humans, which combined with the lack of a licensed inhibitor, leaves a gap in our knowledge and understanding of this serpin in the intricate mechanisms of this complex disease.

Perioperative DVT has been linked to elevated levels of circulating PAI-1 (29). A higher incidence of DVT (46) and venous thrombosis (47) has been noted in Asian Indian patients harboring the 4G polymorphism, leading to a suggestion that it be included in all laboratory testing panels for thrombophilia. Similar studies in white Caucasian populations have described association of the 4G polymorphism with idiopathic DVT and inherited thrombophilia (48). More recently, preoperative plasma PAI-1 has been revealed as an independent risk factor for the onset of DVT in patients undergoing total hip arthroplasty (49). Elevated levels of plasma PAI-1 largely account for delayed clot lysis times in healthy individuals and are associated with first incidence of venous thrombosis (50). These lines of evidence highlight the importance of this serpin in predisposing individuals to a hypofibrinolytic state, which is directly linked to an increased frequency of venous thrombosis. Yet, the exact mechanisms underpinning this pathophysiological process and cellular source of PAI-1 remain to be elucidated.

## Infection and Inflammation

PAI-1 is a positive acute phase protein that is dramatically elevated in the proinflammatory state, such as acute tissue injury, sepsis, and inflammation. The role of PAI-1 in this context is primarily considered a protective mechanism to limit dissemination of pathogens and promote tissue repair. Augmented levels of PAI-1 in non-typeable *Haemophilus influenzae* infection are associated with bacterial clearance and shortening of the disease duration (51). Pharmacological inhibition of PAI-1 in a *Pseudomonas aeruginosa* pneumonia mouse model attenuates neutrophil migration, thereby dampening the innate immune response (52). PAI-1 modulation of neutrophil migration has also been demonstrated in *Escherichia coli* infection (53). However, aberrant activation of this defense mechanism produces a hypofibrinolytic state which promotes thrombotic complications.

Sepsis occurs due to overreaction of the host defense mechanism, most commonly in response to bacterial infection, but can also be caused by viral and fungal pathogens. Sepsis leads to enhanced exposure of the coagulation protein, tissue factor, inciting fibrin deposition and microthrombi throughout the vasculature (54). Multiorgan failure is a frequent complication in sepsis patients, and the development of disseminated intravascular coagulation (DIC) is a major contributor (54) and is associated with aberrant thrombin generation. Endothelial dysfunction induces release of proinflammatory cytokines (55, 56) which combined with augmented levels of thrombin provoke PAI-1 synthesis (Figure 1B). Endothelial cells produce enhanced levels of PAI-1 in response to C-reactive protein (CRP) (57, 58) (Figure 1B), which is a proinflammatory marker in critically ill patients such as sepsis patients (59–61). A recent meta-analysis has reported PAI-1 as a predictor of disease severity in sepsis and overall mortality (62), but the prognostic value of this biomarker in disease progression requires further attention.

A hypofibrinolytic state has been observed in multiple viral infections and is associated with elevated PAI-1 levels (63–67). Most recently, elevated PAI-1 and tPA antigen levels have been described in patients infected with severe acute respiratory syndrome coronavirus 2 (SARS-CoV2) which causes coronavirus-19 disease (COVID-19) (64, 68). However, the net effect of the increased PAI-1 and tPA levels may differ between patients, with variations in this axis being attributed to both a hypo- (64) and hyperfibrinolytic (68) phenotype. In the severe acute respiratory syndrome coronavirus (SARS-CoV) epidemic in 2002 and 2003, the hypofibrinolytic state was attributed to overexpression of PAI-1 which inhibited plasminogen activator activity causing persistence of fibrin deposition (63, 69).

Several proinflammatory cytokines significantly augment PAI-1 synthesis (70–72). Interleukin-6 (IL-6) is an acute-phase inflammatory protein that has been reported to significantly increase PAI-1 and tPA antigen (73). Cytokine release syndrome (CRS) is an acute systemic inflammatory response that can be triggered by various infections and can be observed in sepsis and acute respiratory distress syndrome (ARDS). Endothelial IL-6 trans-signaling promotes IL-6, IL-8, and monocyte chemoattractant protein-1 (MCP-1) and PAI-1 synthesis (55). Inhibition of this trans-signaling circuit by the IL-6R antagonist, tocilizumab, has recently been shown to reduce PAI-1 expression in a small study of COVID-19 patients (55), and is now a recommended treatment for ICU patients after improved outcomes in patients on the Remap-Cap trial (74).

Tumor necrosis factor-alpha (TNF-α), acting *via* NFκB, is a strong stimulator of PAI-1 expression (56) (Figure 1B). TNF-α is an important regulator of PAI-1 expression in adipose tissue, and neutralizing TNF-α significantly reduces both plasma and adipose tissue levels (75, 76). Several studies support the hypothesis that TNF-α may be responsible for expression of TGF-β (75, 77), another major stimulant of PAI-1 biosynthesis (77–80). Elevated levels of PAI-1 in turn block conversion of latent transforming growth factor beta (TGF-β) contributing to a self-regulation mechanism (80). The affiliation between proinflammatory cytokines and heightened PAI-1 synthesis provides a definitive link between this serpin and the inflammatory response.

## Metabolic Syndrome

Metabolic syndrome (MetS) is a multifaceted disorder that encompasses several conditions that considerably elevate the risk of CVD and T2DM. Definitions vary, but in general, diagnosis of MetS requires individuals to meet at least three of the following criteria (81, 82);

- Abdominal obesity
- Dyslipidemia—elevated triglycerides and apolipoprotein B and low levels of high-density lipoprotein (HDL)
- Hypertension
- Hyperglycemia
- Insulin resistance

Whether the clustering of these conditions elevates an individual's risk over that of a single disorder is a matter of ongoing debate (83, 84). Nonetheless, given the prevalence of MetS worldwide and the fact that this cluster of risk factors predicts CVD in multiple settings, it is clear that we require a stronger understanding of the pathophysiology to develop predictive tools and improve therapeutic options.

Abdominal obesity is an essential criterion in the development of MetS (85, 86). Adipose tissue is an endocrine organ composed of multiple cell types, that secrete adipokines of diverse biological function, such as adiponectin (87–90), leptin (91), and various cytokines, including IL-6 and TNF-α. PAI-1 is a known adipokine (8, 92–94). Adipokine secretion is dependent on the location of the fat store in the body and the composition of cells comprised within the adipose tissue. A correlation between MetS and PAI-1 levels was established in the late 1980s (95). Elevated levels of PAI-1 in individuals with MetS has been demonstrated using criteria defined by both the World Health Organization (WHO) (96) and the National Cholesterol Education Program Expert Panel on Detection, Evaluation and Treatment of High Blood Cholesterol in Adults (NCEP-ATPIII) (97). Elevated levels of PAI-1 in humans predict incidence of MetS in two prospective studies (98, 99). It is well-established that PAI-1 can predict the risk of future CVD (100) and onset of T2DM (101). Together, these data have led to the interpretation that PAI-1 is a true component of MetS (102) and could be an important clinical criterion for development of future CVD (103).

### Obesity

Obesity is a global epidemic (104) that is inextricably linked with increased risk of CVD, including arterial, venous, and microvascular thrombosis (105, 106); DVT (107, 108), coronary thrombosis (105, 106), pulmonary embolism (PE) (108, 109) and stroke (110). Obesity-related thrombosis is linked to decreased fibrinolytic activity (111–119) which can be largely attributed to escalating levels of plasma PAI-1 antigen and activity (112, 120). Increased PAI-1 synthesis by adipocytes in response to protractedly elevated levels of TNF-α, insulin, and TGF-β is primarily responsible (112, 114, 120–122) (Figure 1B). Intriguingly, elevated tPA levels have been reported in studies on MetS (96, 97) and obesity (114, 123), which could be the result of impairment of the endothelium; however, the dominant phenotype is hypofibrinolysis.

Plasma PAI-1 significantly correlates with a variety of adiposity measures, including body mass index (BMI), waist-to-hip ratio, total fat mass, and visceral and subcutaneous adipose tissue (124–126). The insulin resistance atherosclerosis study (IRAS) was the first to report that PAI-1 antigen and activity positively correlate with BMI (*r* = 0.314/0.425, respectively) (127). Adipocytes from obese humans harbor twice as much PAI-1 mRNA resulting in a ≥six-fold increase in secretion of PAI-1 and plasma PAI-1 activity compared with lean individuals (8). Weight loss in obese subjects reduces plasma PAI-1 (122, 125, 128), indicative that circulating levels are directly related to the degree of adipose tissue. In line with this, pharmacological inhibition of plasma PAI-1 in animal models results in weight loss, as well as a reduction in adipose tissue and adipocyte volume (19, 129–131). The number and size of lipid-containing vesicles in adipocytes are also decreased, as well as plasma glucose and triglyceride levels and insulin resistance (129, 132, 133). These data indicate that adipocyte-derived PAI-1 functions in an autocrine role, with one study indicating that PAI-1 inhibition limits differentiation of preadipocytes into mature adipocytes (129, 134).

Interestingly, PAI-1 synthesis is not uniform, with adipocytes from visceral fat depots harboring significantly more PAI-1 mRNA than subcutaneous or femoral fat depots (92, 135). Indeed, visceral fat has been suggested as a determinant of PAI-1 activity in overweight and obese women (136). To fully exploit PAI-1 as a biomarker of MetS, and future CVD, it may be necessary to correlate this serpin with additional factors such as visceral fat levels, rather than more general measurements of BMI.

### Type 2 Diabetes

T2DM is intrinsically linked to obesity (92, 137–139), and elevated levels of PAI-1 are strongly correlated with insulin resistance (137, 140), impaired glucose tolerance (137, 140), and T2DM (141, 142). Many studies have reported strong associations between PAI-1 and development of T2DM (143–150). Furthermore, lifestyle and pharmacological interventions to manage diabetes have been shown to decrease circulating plasma PAI-1 levels (151–153). Indeed, PAI-1 activity is significantly reduced upon treatment with the antidiabetic drug, metformin, with a corresponding improvement in glycemic control and reduction in insulin resistance (152).

The IRAS study revealed that PAI-1 was a reliable predictor for developing T2DM, despite adjustments for adiposity, body fat distribution, and insulin sensitivity in patients (*p* = 0.002) (101). Interestingly, this study also found that PAI-1 activity was increased, but not correlated with insulin concentration, in plasma from non-obese children with T2DM parents (101). Various murine models have determined that PAI-1 plays a pivotal role in development of insulin resistance (132, 133). A mouse model of diet-induced obesity was used to study the relationship between PAI-1, obesity, and insulin in PAI-1 deficient PAI-1^−/−^ and wild type (WT) mice (132). Obesity and insulin resistance that developed in WT mice in response to a high-fat diet was prevented in PAI-1^−/−^ mice (132). Furthermore, PAI-1^−/−^ mice showed increased resting metabolic rates and total energy expenditure, compared with WT. Treatment of WT mice with an angiotensin type I receptor antagonist reduced PAI-1 levels, attenuated diet-induced obesity, hyperglycemia, and hyperinsulinemia (132). Genetically obese (ob/ob) mice deficient in PAI-1 weigh significantly less than those with normal PAI-1 levels; as a result, these mice demonstrate a significant improvement in hyperglycemia and hyperinsulinemia (133). Intraperitoneal glucose administration markedly augments serum insulin levels in WT ob/ob mice; however, the increase in PAI-1^−/−^ mice was dramatically reduced (133). *In situ* hybridization studies revealed that TNF-α expression was significantly reduced in PAI-1^−/−^ ob/ob mice compared with WT ob/ob mice (133). Together with the well-documented role of TNF-α in stimulating PAI-1 expression (75), this study exposes a complex reciprocal relationship between PAI-1 and TNF-α which merits further study.

Insulin directly stimulates PAI-1 synthesis and secretion from adipocytes (154) (Figure 1), a process which is upregulated in hyperinsulinemia and hyperglycemia (154, 155). Glucose also upregulates PAI-1 expression in vascular smooth muscle cells, endothelial cells, and adipose tissue (156–159). Clinical studies consistently demonstrate a strong correlation between plasma PAI-1 and insulin resistance (154, 160); however, cause or consequence is less clear, that is whether elevated PAI-1 is a result of insulin resistance or if it occurs independently. The role of TNF-α and TGF-β in stimulating PAI-1 expression in adipose tissue (76, 154) suggests that the increase in plasma PAI-1 and insulin resistance may be bi-directional.

There is evidence to support that chronic inflammation and insulin resistance are linked (101, 143). It is hypothesized that this may be due to increased expression of proinflammatory cytokines, namely IL-6 and TNF-α, from adipose tissue (161, 162) which in turn can stimulate acute phase proteins, including PAI-1 (13, 163). Furthermore, several studies (143, 164, 165) have reported that tPA antigen and activity are associated with developing T2DM, and tPA and PAI-1 antigens are strongly correlated in plasma (166). Despite the concordant increase in tPA and PAI-1, a hypofibrinolytic state prevails in T2DM individuals.

### Hypertension

There is accumulating evidence implicating PAI-1 in the development of hypertension (167), and plasma PAI-1 is associated with several risk factors for hypertension, including obesity (168, 169), insulin resistance (140, 169), and inflammation (70) as discussed above. Genetic ablation of PAI-1 protects against hypertension and perivascular fibrosis induced by nitric oxide synthase (NOS) inhibition (170, 171). NOS plays a key role in regulating vascular tone and remodeling of the vessel wall (172–174), and inhibition of NOS induces progressive hypertension and vascular fibrosis (175–177). Furthermore, inhibition of PAI-1 with a novel small molecule inhibitor (PAI-039) protects a mouse model against angiotensin II-induced aortic remodeling and cardiac fibrosis (178).

Human studies indicate a direct correlation between plasma PAI-1 and hypertension and its associated conditions (179–185), such as arterial stiffness (186) and atherosclerosis (187). Interestingly, the 4G allele for PAI-1 is associated with increased systolic, diastolic, and mean arterial blood pressure (188), indicative of a direct link between plasma PAI-1 and blood pressure. A study examining two longitudinal cohorts of American Indians revealed that baseline PAI-1 is predictive of hypertension independent of other variables (189). Participants with the highest concentration of PAI-1 (>58 ng/ml) had a 63% increased risk of hypertension compared with those in the lowest group (<33 ng/ml) (189). A similar prospective study, the Framingham Offspring Study, confirmed that a higher concentration of plasma PAI-1 was associated with an increased risk of hypertension (odds ratio = 1.28) (190). Despite plasma PAI-1 correctly predicting the risk of hypertension in human studies, it did not provide a significant advantage over conventional risk factors, such as fasting glucose, alcohol consumption, BMI, cigarette smoking, or C-reactive protein (189). Given the close relationship of PAI-1 with the RAS system and the documented increase in the levels of PAI-1 in hypertension, the mechanisms underpinning this relationship warrant further investigation.

## Potential Therapeutic Options and Discussion

**Figure 2:**
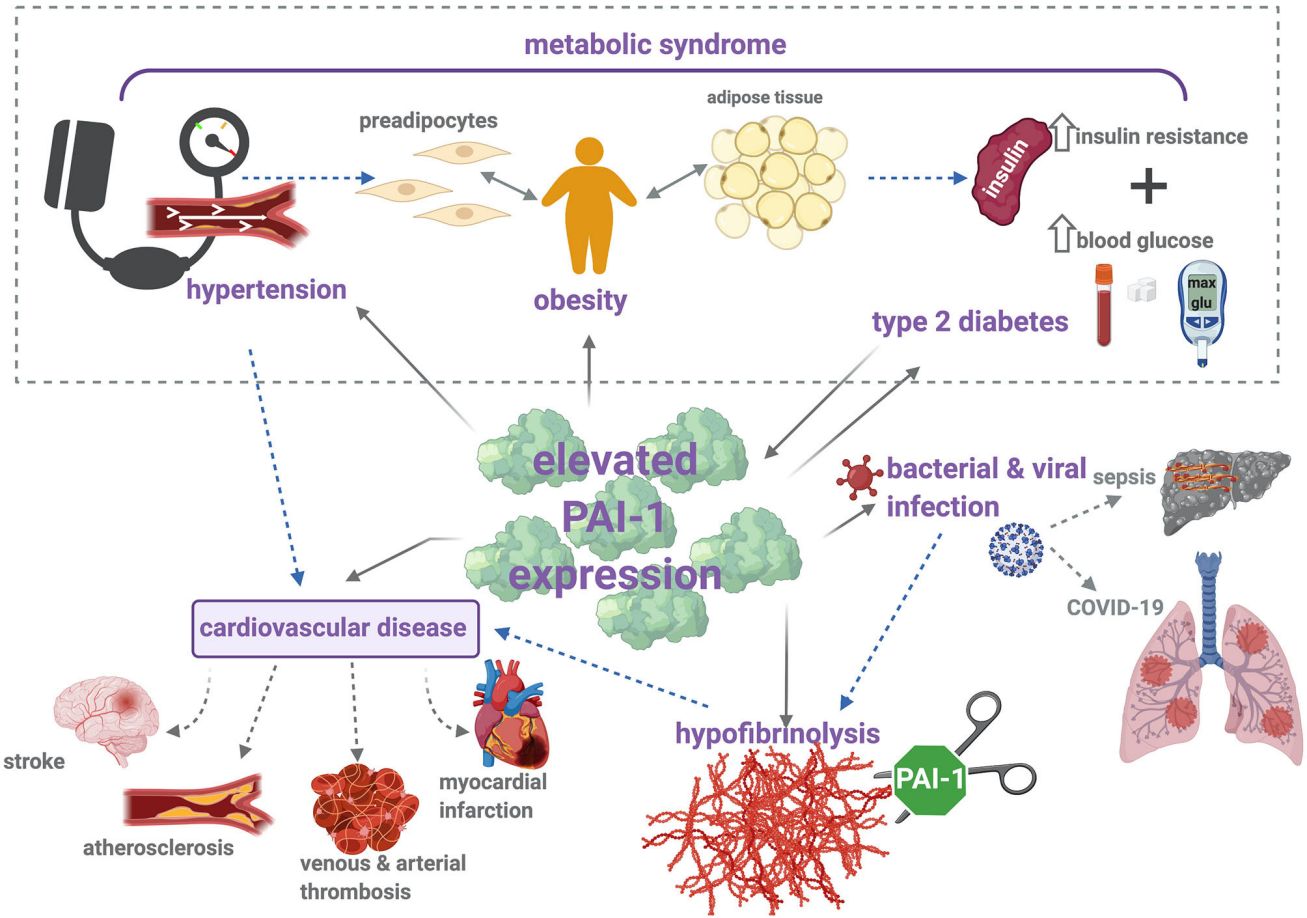
PAI-1 modulates thrombosis and inflammation *via* multiple pathophysiological mechanisms. Metabolic syndrome is characterized by increased insulin resistance, obesity, and hypertension, which contribute to elevated risk of cardiovascular disease (CVD). Increased levels of PAI-1 antigen and activity are positively associated with hypertension, obesity, type 2 diabetes (T2DM), and CVD. Briefly, elevated levels of PAI-1 antigen and activity occur in obesity, which is a known risk factor for CVD and T2DM. Strong correlations between increased PAI-1 and development of T2DM have been identified, including increased insulin resistance and impaired glucose tolerance. Conversely, insulin and glucose can stimulate PAI-1 secretion from adipose tissue. Elevated levels of PAI-1 attenuate plasmin formation and downregulate fibrin degradation. This hypofibrinolytic state provokes thromboembolic complications, including stroke, atherosclerosis, myocardial infarction, and venous and arterial thrombosis. Hypofibrinolysis and elevated PAI-1 levels have been associated with bacterial and viral infections, including sepsis and COVID-19. Sepsis is characterized by the development of disseminated intravascular coagulation (DIC), a major contributor to resulting organ failure. In COVID-19 patients, disruption between coagulation and fibrinolysis leads to fibrin deposits in the lung parenchyma and thrombosis. Solid arrows represent (patho)physiological processes that arise as a result of increased PAI-1 levels, and dotted arrows illustrate the links between each of these individual pathologies.

The driving force of PAI-1 in thrombosis, inflammation, and metabolic syndrome is evident (Figure 2). In addition, this serpin functions in a variety of pathophysiological processes, outwith the subject matter of this review, including wound healing (191), cardiac fibrosis (192), cancer (193), and senescence (194). These numerous roles underscore the potential of PAI-1 as an attractive therapeutic target; nevertheless, to date, no PAI-1 inhibitors have been approved for clinical use.

Small molecules, peptides, monoclonal antibodies, and antibody fragments have all been used to modulate PAI-1 activity by interfering at different stages of the PAI-1/plasminogen activator interaction [(195–199), reviewed in detail by (200)]. A number of clinically approved drugs indirectly reduce plasma PAI-1; these include insulin sensitizing agents for management of T2DM, such as metformin, and ACE inhibitors (used to treat hypertension) (201). However, these drugs have been studied in experimental models (202–205), and as yet, there is limited information available from human studies.

Drugs targeting PAI-1 in the experimental phase have produced promising results (206–208). A potent neutralizing diabody to PAI-1 and activated thrombin activatable fibrinolysis inhibitor (TAFIa) rapidly enhances clot breakdown (207). Simultaneous inhibition of PAI-1 and TAFIa may improve current thrombolytic therapy; e.g., co-administration with tPA thereby permitting a lower dose and thus enhancing its safety profile (207, 209). Tiplaxtinin, a PAI-1 antagonist, prevents venous thrombosis, angiotensin II-induced atherosclerosis, and obesity in a ferric chloride-induced vascular injury model in rats (206). More recently, a nanobody to PAI-1 has been developed that selectively stabilizes the active form of PAI-1, which may be used as a diagnostic or analytical tool (195, 208).

Other drugs that elicit pharmacological inhibition of PAI-1 have reached phases 1 and 2 clinical trials. A small molecule inhibitor, TM5614, is currently being trialed in a single-center, randomized controlled trial for high-risk patients hospitalized with severe COVID-19 and requiring oxygen (210). Another PAI-1 inhibitor, ACT001, is currently in phase 1 clinical trials for treatment of glioblastoma, the most aggressive primary malignant brain tumor in adults (211, 212).

This review predominantly focuses on the role of PAI-1 in the thrombosis-inflammation axes and associated diseases (Figure 2). It is evident that a proinflammatory state, whether it arises from infection, vascular thrombotic complications, a condition such as MetS, or its associated cluster of diseases spark a dramatic elevation in plasma PAI-1. The underlying motive for this can be attributed to the many cytokines and proteins that can elicit PAI-1 synthesis, thereby inextricably bridging these conditions with this complex serpin. The cellular source of PAI-1 may vary according to the disease processes, e.g., of platelet or endothelial origin during infection or venous thrombosis but of adipose tissue origin in obesity. Many of the conditions described herein predominantly affect the aging population, and it is noted that there is also a clear link between circulating PAI-1 levels and age (213). These observations further confound the relationship between elevated PAI-1 and thromboinflammation, leading to cardiovascular complications. This review serves as an aide-mémoire on the consequences of PAI-1 elevation and highlights the utility of this serpin as a potential therapeutic target in the treatment of various pathological conditions which are associated with a hypofibrinolytic state and development of thromboembolic diseases.

## Author Contributions

GM and CW researched the data, wrote, and revised the manuscript. NM conceived the article, wrote the article, and edited the article. All authors contributed to the article and approved the submitted version.

## Conflict of Interest

The authors declare that the research was conducted in the absence of any commercial or financial relationships that could be construed as a potential conflict of interest.
